# Six Letters with Answers

**Published:** 1881-04

**Authors:** 


CINCINNATI, Ohio, March 1, 1881.

DR. UP DE GRAFF:

My little daughter, aged 14, has just passed through a severe sickness of scarlet fever, and now is troubled with a terrible ear-ache, which nothing that our physician or ourselves have done relieves. The ear is hot and swollen, and a very tender and red place shows just behind the ear. The doctor says it will break there. Is there nothing we can do to relieve this terrible suffering? * * * *

MRS. O. B. H.

To this mother we wrote as follows, and print it here for the information of others: An abscess has formed in the mastoid cells, as often happens in the otorrhœa accompanying scarlet fever. The pain can be relieved and the progress of the disease cut short by freely opening the prominence back of the ear. Apply to some capable surgeon and have the operation performed. This will save your child much suffering.

---

WAYLAND, N. Y., Feb. 24, 1881.

THAD S. UP DE GRAFF, M. D.:

*Dear Sir.*—What do you think of rotten apples applied to the throat, and sulphur given in milk for diphtheria? MRS. R.

What do we think? Well, we should say it would be a soft, sticky, stinking mess to apply to a well person's throat—and all the more so to a sick one's—without possessing any other qualification that we can just at the moment think of. The milk and sulphur would be a harmless sort of a mixture—but the milkmen, up this way are accused of putting chalk in their milk. Upon the whole, if milk *must* be adulterated, we prefer the chalk to the sulphur—it looks better and wouldn't make a fellow think of his "hereafter" quite so much.

As to the diphtheria, you had better call a physician.

---

LEXINGTON, Ky., March 9th, 1881.

DR. THAD S. UP DE GRAFF:

*Dear Sir.*—I read your reports, in the microscopical journals, of the doings of the Elmira Microscopical Society, and therefore take the liberty of asking whether you are a believer in evolution. Are you? J. D. S., M. D.

Yea, verily! Believest thou in "eternal damnation?" Not that we care a fig whether you do or not, but to simply gratify a sort of vague desire to ask questions. It's a funny and innocent amusement you know.

---

PINE VALLEY, N. Y., March 12th, 1881.

DR. UP DE GRAFF:

The spectacles I bought of you when you called at my house last week, don't suit. They hurt my eyes and everybody says I paid you too much for them. As you said you would refund the money if I did not like them, please send my ten dollars back and I will send you the spectacles. MRS. ———

That's fair. We're very much obliged for the confidence you reposed in us, but are immediately humiliated to think you recognized *us* in a miserable, lying, hump-backed, swindling, spectacle pedlar. That is just a trifle tough. That chap is entirely welcome to your ten dollars—as far as we are concerned, but we have a kind of lingering desire that he would either change his name or his occupation—we don't care which.

---

WILLIAMSPORT, Pa., Feb. 10th, 1881.

DR. UP DE GRAFF:

I send you by express, charges paid, a bottle of water taken from a well in this vicinity, among the drinkers of the water of which, in six months, nine deaths have occurred. Four adults and five children have died from typhoid fever or diphtheria. I suspect the water, particularly as I notice a dirty sediment from standing over night. Yours, O. J.

The water contains living vorticellæ, paramaceum, monads, and bacteria. The well, manifestly has a communication with some cesspool in the neighborhood, or nasty surface water has ingress to it. Such creatures only abound in decomposing animal and vegetable matter. The well is sufficient cause for the diseases mentioned. Abandon it.

---

VINCENNES, Ind., Feb. 26, 1881.

EDITOR BISTOURY:

Please inform me where I may obtain the eucaliptus globulus. I desire to try it on some cases of intermittent fever that do not behave well under quinia.

Yours, J. D., M. D.

Write to Parke, Davis & Co., whose address will be found in our advertising columns.

